# Respiratory infections in children and adolescents in Germany during the COVID-19 pandemic

**DOI:** 10.25646/11437

**Published:** 2023-06-14

**Authors:** Udo Buchholz, Ann-Sophie Lehfeld, Kristin Tolksdorf, Wei Cai, Janine Reiche, Barbara Biere, Ralf Dürrwald, Silke Buda

**Affiliations:** 1 Robert Koch Institute, Berlin Department of Infectious Disease Epidemiology; 2 Robert Koch Institute, Berlin Department of Infectious Diseases

**Keywords:** RESPIRATORY INFECTIONS, COVID-19 PANDEMIC, GRIPPEWEB, RSV, INFLUENZA VIRUS, RHINOVIRUS, SARS-COV-2

## Abstract

**Background:**

Before the COVID-19 pandemic, acute respiratory infections (ARIs) in children were mainly characterised by three pathogens: respiratory syncytial viruses (RSV), influenza viruses and rhinoviruses. The impact of the COVID-19 pandemic and the measures taken in Germany (especially until the end of 2021) on the incidence of ARI in children and adolescents aged 0 to 14 years and the pathogens causing them has not yet been comprehensively analysed.

**Methods:**

The evaluation is based on data from population-based, virological and hospital-based surveillance instruments up to the end of 2022.

**Results:**

After the onset of the COVID-19 pandemic in early 2020, ARI rates remained almost consistently below prepandemic levels until autumn 2021, with only rhinoviruses continuously continuing to cause ARI. Only when the Omicron variant became predominant in 2022, there were measurable COVID-19 rates at population level in children, although COVID-19 hospitalisation rates remained comparatively low. RSV and influenza waves were initially absent and then occurred ‘out of season’, but were more severe than usual.

**Conclusions:**

While the measures taken were effective in inhibiting the number of respiratory infections for almost 1.5 years, moderately frequent but rather mild COVID-19 cases occurred when measures were lifted. When Omicron emerged in 2022 COVID-19 became moderately frequent but led predominantly to mild illnesses. For RSV and influenza, the measures resulted in changes in their annual timing and intensity.

## 1. Introduction

Acute respiratory diseases are among the most common infectious diseases in childhood. They are mostly caused by viral pathogens with differences in the age group. Before the emergence of SARS-CoV-2 respiratory syncytial viruses (RSV) (Info box 1), influenza viruses (Info box 2) and rhinoviruses (Info box 3) dominated the epidemiology of acute respiratory diseases in children.

**Figure 1:**
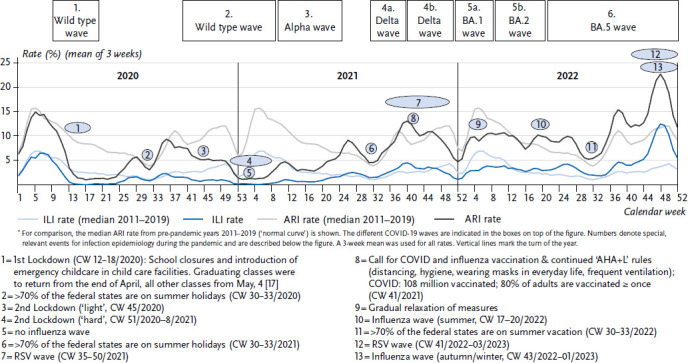
Rates of acute respiratory illnesses (ARI) and influenza-like illnesses (ILI) in children aged 0–14 years in 2020–2022^*^ Source: GrippeWeb

The activity of acute respiratory diseases in children had a distinct, typical annual course before the COVID-19 pandemic (faint grey curve in Figure 1). Only in the first three to four months of the year some variation in the activity of acute respiratory illnesses could be observed. Seasonal peaks were usually reached during this period of time, primarily influenced by seasonal influenza and RSV waves that were more or less severe. After this peak phase followed normally a downward trend to the lowest level of the year around calendar week (CW) 32, when the summer holiday season was in full swing. Thereafter, there was usually a relatively steep rise with an early autumn peak, a short decline at the time of the autumn holidays and another peak just before the end of the year. Overall, the rate of acute respiratory illnesses was significantly higher in October to March as compared to spring and summer.

In December 2019, a cluster of pneumonia cases (including fatalities) with initially unknown causes occurred in Wuhan, China. The causative agent was identified as beta-coronavirus [12]. The virus and the disease it caused were later named SARS-CoV-2 and COVID-19, respectively [13]. Starting in January 2020 the first COVID-19 cases were diagnosed in Germany [14], and on 11 March 2020, the World Health Organization (WHO) declared the outbreak to be a pandemic [15].

Many countries around the world took – mostly – multiple complementary so-called non-pharmaceutical measures during the course of the pandemic, such as temporarily cancelling events with more than 1,000 participants [16]. In Germany, along with the first lockdown of the COVID-19 pandemic in week 12/2020 and the second lockdown in week 51/2020, daycare centres and schools were also closed. After six and eight weeks, respectively, the facilities were successively opened again [17], partly with restricted access and/or assigned fixed/divided groups.

Thus far, the impact of the COVID-19 pandemic on respiratory infections in children in Germany and the pathogens that triggered them have not been comprehensively analysed for the first three years of the pandemic, i.e. 2020−2022. There is a need to investigate to what extent the measures taken for COVID-19 and changes in everyday life may have changed the incidence of respiratory illnesses, or which pathogens have led to respiratory illnesses despite the measures taken. Furthermore, it is unclear how long possible changes in the incidence rates of pathogens lasted and whether or not there were any ‘catch-up effects’ later in the pandemic.

In this paper we assess and discuss the incidence of acute respiratory infections in children and adolescents aged 0 to 14 years during the COVID-19 pandemic, the pathogens that caused acute (and severe) respiratory infections in children, the pathogens that circulated concurrently, and the incidence of dual infections for the period of 2020–2022. We will focus on influenza viruses, RSV, rhinoviruses and, lastly, SARS-CoV-2.

**Info box 1**

**Respiratory syncytial virus (RSV) infections**

RSV infections occur seasonally, usually with a peak in incidence just before or after the turn of the year [1]. Although they can occur at any age, they clinically manifest as a typical illness in infancy and early childhood. By the second year of life, virtually all children have experienced a first infection with RSV, a large proportion even two of them [2]. The disease may be confined to the upper respiratory tract, but can also affect the lower respiratory tract (bronchiolitis, pneumonia or tracheobronchitis), especially in infants in their first months of life [3]. Febrile courses are frequent.

**Info box 2**

**Influenza**

Influenza (common cold, flu) is caused by influenza viruses who are sub-divided into two types (influenza A and influenza B), with the influenza A viruses comprising two subtypes A(H1N1) and A(H3N2). Prior to the COVID-19 pandemic, influenza waves occurred seasonally, unless a novel influenza virus emerged in the population and led to pandemic spread, as was the case during the 2009 influenza pandemic caused by influenza virus A(H1N1)pdm09. Transmission of influenza virus from animals to humans occurs very rarely, but if so then through poultry or pigs. These cases are called zoonotic infections. Accordingly, a distinction is made between seasonal, pandemic and zoonotic influenza.

Influenza affects all age groups. At the age of eight most children had at least one influenza A infection as can be shown through serological studies [4]. Between the child age groups either the 0- to 4-year-olds or the 5- to 14-year-olds have the highest incidence rate, depending on the season [5]. The incidence rate generally decreases with increasing age, but the probability of severe illness or illness requiring hospitalisation increases [5, 6].

## 2. Methods

### 2.1 Data basis

The data for the results shown here focus on children and adolescents (hereinafter ‘children’) between 0 to 14 years of age. For illustrative purposes, data from older age groups are occasionally included for comparison. We used data from the following surveillance instruments:

1. the population-based survey instrument GrippeWeb,
2. the Virological Surveillance of the German ‘Working Group for Influenza’ (Arbeitsgemeinschaft Influenza (AGI)),
3. the ICD-10 code-based hospital surveillance of severe acute respiratory infections (ICOSARI).

The three systems have been described in detail by Goerlitz et al. [18]. GrippeWeb is an online portal of the Robert Koch Institute (RKI), where any person from the general population in Germany with a minimum age of 16 years can register. Parents with children younger than 16 years can report information on acute respiratory diseases for their children as well. Thus the entire age spectrum is covered. Participants report weekly whether or not they have had a new acute respiratory illness in the previous week. If this is the case further information on symptoms, among other items, is requested. For the analyses, acute respiratory illnesses (ARIs) are defined as acute illnesses of the respiratory tract with at least one of the three symptoms fever, sore throat or cough is reported. All ARIs with fever plus sore throat or cough are defined as ILI (influenza-like illness). Thus, ILI are a subgroup of ARIs. ‘Non-ILI’ are ARIs that do not meet the ILI definition, thus ILI + non-ILI = ARI. To obtain population-based rates, data are adjusted for federal state, age group and sex. Current results from GrippeWeb are published weekly on the RKI website. In the analysis period 01. Jan 2020 – 31. Dec 2022 we received weekly reports from about 7,500–10,000 adults and children.

**Info box 3**

**Rhinoviruses**

More than 100 so-called serotypes of rhinoviruses are known [7]. Rhinovirus infections are causative for the majority of colds in all age groups and show no pronounced seasonality [7, 8]. They are frequently identified as triggers of respiratory illnesses in the summer months. Some authors attribute an important role in childhood pneumonia to rhinoviruses as well [9, 10], although rhinovirus-associated pneumonia tends to be milder in contrast to, for example, RSV- or influenza-associated pneumonia [11]. In addition, rhinoviruses have been linked to exacerbations of asthmatic lung disease [10, 11].

The **Virological Surveillance** of the ‘Working Group for Influenza’ (Arbeitsgemeinschaft Influenza (AGI)) is a task of the National Reference Laboratory for Influenza (NRLI) at the RKI. For this purpose, a subgroup of phyisicians participating in the AGI take samples from the nose or throat of patients with acute respiratory illness. In addition, phyisicians collect and provide information about these patients’ respiratory symptoms. It is therefore possible to distinguish between patients with ILI and non-ILI. Samples and questionnaire are sent together to the NRLI, where they are tested for viral respiratory pathogens. These include SARS-CoV-2, influenza viruses, rhinoviruses and RSV.

In the **hospital surveillance project ICOSARI** the RKI works with approximately 70 sentinel hospitals throughout Germany within the framework of a scientific cooperation. Data on severe acute respiratory infections (SARI) are collected and sent to RKI via the network’s data centre. The system covers approximately 5 to 6% of all patients hospitalised in Germany. The data contain ICD-10 codes, with SARI being defined as inpatients with an ICD-10 code between J09 and J22 (influenza, pneumonia, other acute lower respiratory tract infections) in their main DRG diagnosis. Diagnosis-related groups (DRGs) refer to a classification system in which treatment cases are combined into case groups on the basis of certain criteria (ICD-10 codes). In addition, specific diseases such as COVID-19, influenza and RSV can be recorded via specific codes in the principal or secondary DRG diagnosis [19].

We analysed the different COVID-19 waves with dominance of certain SARS-CoV-2 variants (VOC – variants of concern) in the general population:

► Wild type from CW 10–20/2020 and CW 40/2020–08/2021,
► Alpha from CW 09–23/2021,
► Delta (a) from CW 31–39/2021,
► Delta (b) from CW 40–51/2021,
► Omicron BA.1 from CW 52/2021–08/2022,
► Omicron BA.2 from CW 09–21/2022,
► Omicron BA.5 from CW 22/2022 until the end of the study period in CW 52/2022 [20, 21].

The influenza wave in the general population lasted in 2020 from CW 02–12 [22], in summer 2022 from CW 17–20 [23] and in autumn/winter 2022/2023 from CW 43/2022–CW 01/2023 [24]. The RSV wave in 2021 lasted from CW 35–50 [1], and in the 2022/2023 season it started in CW 41 and lasted until CW 03 [25].

**Info box 4**

**Pathogen-specific ARI rates**

To calculate the pathogen-specific ARI rates, we took into account the fact that ILI and non-ILI can have different positivity rates ((PR) = proportion of positive respiratory samples in all respiratory sentinel samples sent to the NRLI)) for certain pathogens and that the distribution of ILI/non-ILI in the samples may not correspond to the ILI/non-ILI distribution in disease incidence.

However, since the individual parameters are known, the ARI rates for each pathogen can be weighted by ILI/non-ILI. We used the virological surveillance data to calculate the pathogen-specific PR of individuals with ILI and the PR of individuals with non-ILI. In addition, separate ILI and non-ILI rates were calculated from GrippeWeb. In the next step we multiplied the weekly ILI and non-ILI rates from GrippeWeb with the respective weekly pathogen-specific PR of individuals with ILI and non-ILI; then we added both rates then added both rates to obtain the proportion of the ARI rate attributable to a particular pathogen.

We used the following formula:

ILI rate(GW)*PR_ILI_(pathogen X)+non-ILI rate(GW)* PR_non-ILI_(pathogen X)=ARI rate(GW) for pathogen X

GW=GrippeWeb data,

ILI=influenza-like illness,

ILI rate(GW)=ILI rate calculated from GW data,

PR_ILI_(pathogen X)= positivity rate of individuals with ILI for pathogen X,

PR_non-ILI_(pathogen X)= positivity rate of individuals with non-ILI for pathogen X

Hypothetical example for the calculation of pathogen-specific ARI rates using influenza as an example: The ARI rate in one calendar week was 6%, sub-dividable into 1% ILI and 5% non-ILI. The influenza PR in the same calendar week was 27% for patients with ILI and 12% for non-ILI patients. Thus, the influenza rate for ARI was: 1%*27% (ILI)+5%*12% (non-ILI)=0.27% (ILI)+0.6% (non-ILI)=0.87% (ARI due to influenza). Thus, 0.87% of 6%=14.5% of the ARI rate would be due to influenza.

### 2.2 Analyses and calculations

We used the ARI rate in the population collected via GrippeWeb to calculate rates of ARI attributable to a particular pathogen (pathogen-specific ARI rates; Info box 4). This was done for the group of children (0 to 14 years) and partially also for adults (15 years and older), but also separately for the groups aged 0 to 4 years and 5 to 14 years. This allowed us to compare which pathogen-specific ARI rates were elevated in specific age groups.

We calculated the average number of ARI for consecutive six-month periods, starting with the autumn/winter half-year (CW 40/2017−CW 13/2018) and ending with the spring/summer half-year (CW 14−39/2022) in the age groups 0–4 years, 5–14 years and 15 years and older. For this purpose, we only used the data of GrippeWeb respondents who had consistently or almost consistently submitted their weekly report for the respective six-month period. Specifically, we limited this calculation to all respondents with 90% or higher weekly participation during the respective period.

We also calculated the incidence of co-infections, i.e. simultaneous infection of the same individual with different pathogens. To do this we analysed every sample from the 2020–2022 period that was received at the NRLI as part of their virological surveillance. A co-infection was defined to be present if more than one of the following pathogens was detected: Influenza A(H3N2), influenza A(H1N1)pdm09, influenza B, RSV, rhinovirus, SARS-CoV-2, human coronavirus HKU1, human coronavirus NL-63, human coronavirus OC-43, human coronavirus 229E, parainfluenza virus type 1–4 (PIV) and human metapneumovirus (hMPV). We then compared the actual incidence to an expected incidence (Info box 5).

We assumed statistical significance if the calculated p-value was below 0.05 or if the (95%) confidence intervals did not overlap.

We thus calculated the cumulative number of illnesses hospitalised during a COVID-19 wave or a wave due to RSV or influenza by age groups (0–4, 5–14, 15–34, 35–59, 60–79 years and 80 years and older). For this purpose, we added up for each age group within the respective period the weekly number of SARIs for which the specific pathogen was coded via the ICD-10 code in the main or secondary diagnosis and extrapolated to the whole of Germany.

We used demographic data of the German population of the Federal Statistical Office with data status 31 Dec 2021 to calculate weekly rates, which are presented as a percentage of the population. A rate of e.g. 1% corresponds to an incidence of 1 per 100 residents or 1,000/100,000 residents.

**Info box 5**

**Expected incidence of co-infection**

We calculated the expected incidence of co-infection by two specific pathogens (i.e. simultaneous infection of an individual by two pathogens) for selected annual intervals in which two specific pathogens were present at sufficiently high incidence in the group of children. In order to exclude age effects (e.g. if one pathogen occurs much more frequently in 0- to 4-year-olds than in 5- to 14-year-olds) and since co-infections manifest more frequently in young children, we restricted the calculation to the age group of 0- to 4-year-olds. We multiplied the ARI-positive rate (Info box 4) of one pathogen by the ARI-positive rate of the other pathogen [26]. For example, if the PR of one pathogen was 30% and that of a second pathogen was 20% in a CW, the expected co-infection rate would be 6% (=30%*20%). We assumed that the infections with the individual pathogens occur independently of each other and are evenly distributed throughout Germany. We compared this expected incidence to the actually observed incidence of the respective co-infection.

## 3. Results

### 3.1 Respiratory infections in general and pathogen-specific ARI rates

**Figure 2:**
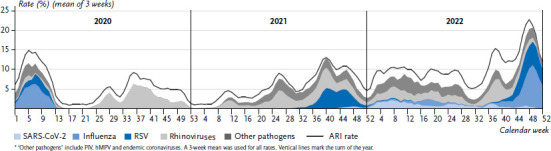
Rate of acute respiratory illness (ARI) and the respective proportions of the various pathogens attributable to the ARIs (areas) in children aged 0–14 years from 2020–2022^*^ Source: GrippeWeb and Virological Surveillance of the Working Group for Influenza (Arbeitsgemeinschaft Influenza)

Figure 1 shows the course of ARI or ILI rates in children up to 14 years of age (recorded by the GrippeWeb portal) in the period from CW 01/2020 to CW 52/2022 compared to pre-pandemic years. Figure 2 shows the proportion of the ARI rate that was caused by the respective pathogens.

At the start of the pandemic (around week 10/2020), ARI activity was roughly the same as expected from the respective weeks in the pre-pandemic years. After the first lockdown (CW 12/2020), both ARI and ILI rates dropped to very low levels. After the end of the first COVID-19 wave (CW 20/2020; [27]) and the gradual relaxation of many containment measures, rhinoviruses began to circulate again in May/June 2020 and the ARI rate in children rose to pre-pandemic levels (Figure 1). From around CW 37/2020 the ARI rate deviated again from the usual pre-pandemic course as it did not reach the usual early autumn peak, but gradually declined to very low levels by the end of the year. In the winter of 2020/2021, the usual wave of influenza in the first three to four months of the year was absent, as was the RSV wave. Rhinoviruses, in particular, continued to cause respiratory illnesses during this period and until the middle of 2021 (Figure 2).

In the early summer of 2021 as well as in the (early) summer of 2022, parainfluenza viruses circulated in children in addition to rhinoviruses, in the spring of 2022 human metapneumoviruses circulated also; the somewhat pronounced circulation of some endemic coronaviruses such as NL63 and OC43 in 2021 and 2022, on the other hand, does not seem to have had a major impact on ARI rates (Figure 2, PIV, hMPV and endemic coronaviruses are summarised under ‘other pathogens’).

The ARI rate in children increased rapidly after the usual low level in the summer (week 32/2021). This was accompanied by the start of a strong RSV wave in CW 35/2021 outside the usual season, to the extent that the early autumn peak of the ARI rate observed in pre-pandemic years was clearly exceeded in CW 36/37. Along with the onset of the fourth COVID-19 wave dominated by VOC Delta, illnesses with SARS-CoV-2 infections became detectable at population level for the first time in children, i.e. about 0.5% of children contracted COVID-19 on a weekly basis (light blue area in Figure 2).

From around week 46/2021 onwards, the ARI rate began to decline steadily again and was below pre-pandemic levels – similar to 2020, but less marked. After the onset of the Omicron wave (subvariants BA.1 and then BA.2 between January and April 2022), COVID-19 affected children to a clearly detectable degree. At least 1 to 2% of children contracted COVID-19 every week (Figure 2) during these four months. Nevertheless, respiratory illnesses caused by rhinoviruses also occurred at least as frequently as those caused by SARS-CoV-2 during this period (light grey area, Figure 2). During that time, the ARI rate did not exceed the values observed in pre-pandemic RSV or influenza waves in the months of January to March.

In May (CW 17–20/2022), there was a mild and short influenza wave, and from May/June 2022 (from about CW 21/2022), the BA.5 subvariant of Omicron also caused weekly illnesses again in children at a level of up to 1.3%. Even at the time of the lowest level of the year (while summer vacations of most federal states were ongoing), ARI activity was somewhat higher than usual (Figure 1). In late summer, rhinoviruses were the first to cause a steep rise in the ARI rate with an unusually high early autumn peak. A few weeks later, early-onset and almost parallel waves of RSV and influenza led to ARI rates in excess of 20% in children (0–14 years), which were exceptionally high not only for this time of year but also in absolute terms. Seasonal peaks reach normally only 18–19% in children. Only in the last weeks of the year did ARI activity decline again.

Overall, the course of the ILI rate (faint blue in Figure 1) in both the pandemic years and pre-pandemic years (2011–2019) roughly paralleled that of the ARI rate, but at a lower level.

**Figure 3:**
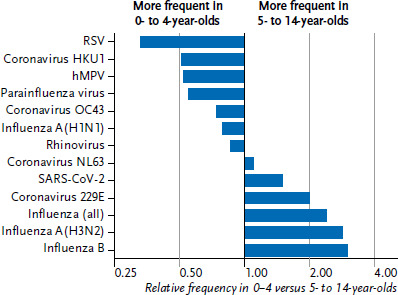
Relative incidence of all ARI due to different respiratory pathogens in 0- to 4-year-olds compared to 5- to 14-year-olds (for the period of 2020−2022; proportion adjusted for ILI and non-ILI); the x-axis is plotted logarithmically Source: GrippeWeb and Virological Surveillance of the Working Group for Influenza (Arbeitsgemeinschaft Influenza)

Over the entire period (2020–2022), about a quarter (25.3%) of ARI in children were due to rhinoviruses, followed by influenza and RSV (Table 1). Comparing the two child age groups, 0- to 4-year-olds were diagnosed with RSV, HKU1 coronaviruses, hMPV and PIV more frequently, while 5- to 14-year-olds were diagnosed with influenza A(H3N2) and influenza B, coronavirus 229E and SARS-CoV-2 more frequently (Figure 3).

### 3.2 Seasonal individual ARI frequency and multiple infections in ARI in different age groups

**Figure 4:**
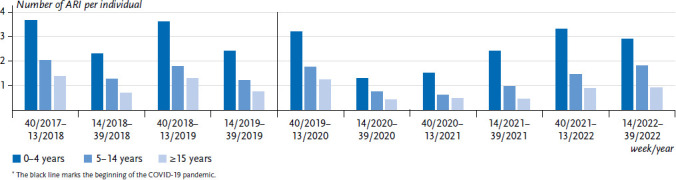
Average number of acute respiratory illnesses (ARI) per person participating in GrippeWeb in the autumn/winter (CW 40 to CW 13) and spring/summer (CW 14 to CW 39) half-years from 2017–2022; age groups 0 to 4 years, 5 to 14 years and 15 years and older^*^ Source: GrippeWeb

The mean number of ARI decreased with increasing age in all half-years from 2017−2022, and was – before the pandemic – about 1.5–2 times higher in the autumn/winter season (CW 40–CW 13) than in the spring/summer half-year (CW 14–CW 39; Figure 4) in all age groups. Children aged 0–4 years had an average of about 3–4 ARI in the autumn/winter half-year, while 5- to 14-year-olds had just under 2 ARI and adults approximately 1.3 ARI. Upon the start of the pandemic, the average number of respiratory infections decreased sharply in all age groups, but then rose again and almost reached the pre-pandemic level again in the autumn/winter half-year 2021/2022 (CW 40/2021-CW 13/2022).

**Figure 5:**
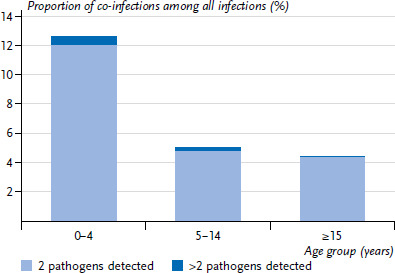
Proportion of all infections in which two or more than two pathogens were detected; pooled for 2020–2022, stratified by age group Source: Virological Surveillance of the Working Group for Influenza (Arbeitsgemeinschaft Influenza)

Dual infections with the viruses examined in the NRLI occurred between 2020 and 2022 mainly in 0- to 4-year-old children, at about 12% (Figure 5). However, infections by more than two of the pathogens examined in the pathogen panel were rare even in this age group. In children aged five years and older, the proportion of co-infections was about 5%.

**Figure 6:**
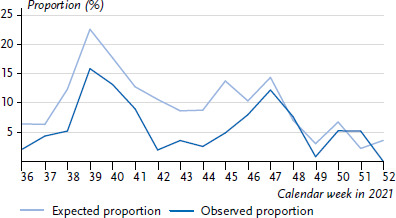
Expected and observed proportion of co-infections by rhinoviruses and RS-viruses in 0- to 4-year-olds, from CW 36 to 52/2021 Source: Virological Surveillance of the Working Group for Influenza (Arbeitsgemeinschaft Influenza) and GrippeWeb

**Figure 7:**
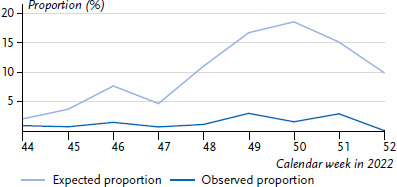
Expected and observed proportion of co-infections by influenza and RS-viruses in 0- to 4-year-olds, from CW 44 to 52/2022 Source: Virological Surveillance of the Working Group for Influenza (Arbeitsgemeinschaft Influenza) and GrippeWeb

RSV and rhinoviruses both circulated concurrently at the end of 2021; the incidence of rhinovirus-RSV co-infections among 0- to 4-year-olds during this period was roughly in line with the expected incidence (Figure 6), while the actually observed incidence of influenza-RSV co-infections at the end of 2022 among 0- to 4-year-olds was below the expected incidence by a factor of 5 (Figure 7). Likewise, rhinovirus-SARS-CoV-2 co-infections did not occur as frequently as expected from theory for 0- to 4-year-olds between CW 01 and CW 16/2022 (not shown).

### 3.3 COVID-19

#### COVID-19 rate in children with symptoms of acute respiratory infection

**Figure 8:**
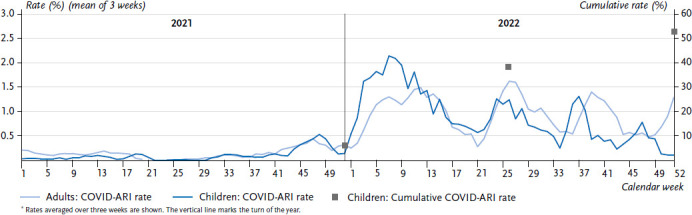
COVID-19 rate in the population calculated from GrippeWeb participants with symptoms of ARI (COVID-ARI rate), stratified by children (0 to 14 years) and adults (15 years and older) from 2021–2022^*^. Squares represent the cumulative (summed up) COVID-ARI rate of children at the end of 2021, mid-year 2022 and end of 2022 (right y-axis) Source: GrippeWeb and Virological Surveillance of the Working Group for Influenza (Arbeitsgemeinschaft Influenza)

The rate of COVID-19 plus ARI (COVID-ARI rate) among children aged 0 to 14 calculated via GrippeWeb and from the data of the virological surveillance remained at a low level, i.e. well below 0.5%, until the fourth wave with the predominant Delta variant. During the Delta (b) wave – towards the end of 2021 – the COVID-ARI rate increased for the first time to about 0.5% (Figure 8). In the first four months of 2022, the predominant subvariants were Omicron BA.1 and BA.2. During this time the COVID-ARI rate in children increased to more than 2.0% and was clearly higher than the COVID-ARI rate in adults. This scenario reversed when Omicron subvariant BA.5 became predominant in two waves with one peak in CW 26/2022 and one in CW 37 (children) or CW 39 (adults). According to this estimate, approximately 6% of 0- to 14-year-old children were affected by COVID-ARI cumulatively from the beginning of the pandemic until the end of 2021, approximately 38% by the end of June 2022 and 53% by the end of 2022 (squares in Figure 8).

#### Severe courses of disease

**Figure 9:**
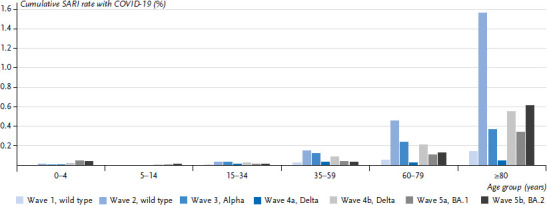
Cumulative rate of severe acute respiratory infections (SARI) with COVID-19 in different phases of the 2020–2022 pandemic and in six different age groups. As the end of the Omicron-BA.5 phase had not yet been defined at the time of the study period of this paper, it was not yet possible to calculate cumulative rates for this phase Source: ICOSARI Hospital Sentinel

The cumulative incidence of severe acute respiratory infections (SARI) diagnosed with COVID-19 was lowest among 5–14 year old children in all phases of the pandemic (Figure 9). Among children between 0 and 4 years of age, the cumulative COVID-SARI incidence was higher than in 5- to 14-year-old children, but clearly lower than among adults aged 60 to 79 years or even older. Among 0- to 4-year-olds and 5- to 14-year-olds, the cumulative incidence was highest during the predominance of Omicron subvariants BA.1 and BA.2 at the start of 2022, at 0.4% (corresponding to 40 COVID-SARI cases per 100,000 residents) and 0.1% (corresponding to 10 COVID-SARI cases per 100,000 residents), respectively.

**Figure 10:**
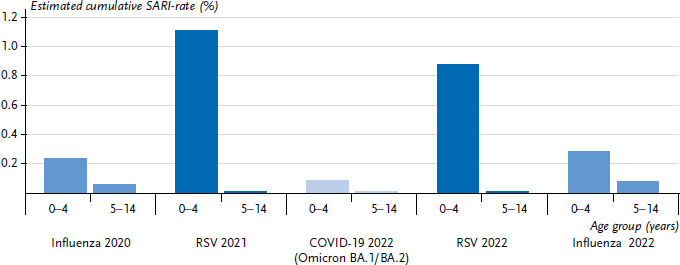
Cumulative rates of SARI with ICD-10-coded influenza diagnosis, RSV diagnosis or COVID-19 in 0- to 4-year-olds and 5- to 14-year-olds, shown for the strongest or only pathogen waves in the period under consideration (2020–2022). Waves were ordered chronologically as follows: Influenza early 2020, RSV 2021, COVID-19 (Omicron BA.1 and BA.2) early 2022, RSV year-end 2022 and influenza year-end 2022 Source: ICOSARI Hospital Sentinel

Figure 10 shows how often children were affected by severe respiratory illness with a COVID-19 diagnosis (i.e. hospitalisation). This is compared to the burden of disease of severe acute respiratory illnesses caused by other pathogens that circulated in 2020–2022 and frequently resulted in SARI. For this purpose, we compared the cumulative rate (incidence per 100 residents) during the Omicron-BA.1/ BA.2 waves to the cumulative SARI rate of the (abbreviated) influenza wave in 2020, the RSV waves in 2021 and 2022, and the influenza wave in 2022 (Figure 10). It was evident that the RSV waves led to the highest cumulative rates in 0- to 4-year-olds (dark blue bars in Figure 10), whereas the influenza waves led to the highest cumulative rates in 5- to 14-year-olds (blue bars in Figure 10). The cumulative rates of the 5- to 14-year-olds are lower than those of 0- to 4-year-olds for all pathogens and in all waves. Whereas an average of 0.5% of 0- to 4-year-old children was hospitalised for RSV in winter between 2014/2015 and 2019/2020 before the pandemic, this increased significantly by a factor of 2.2 to 1.1% in the 2021 RSV wave. Likewise, in 2022 (CW 41–CW 52/2022), the proportion of 0.9% was 1.8-fold higher as compared to an average pre-pandemic RSV wave (the difference is also significant; not shown). Similarly, the influenza wave at the end of 2022 resulted in higher rates of SARI due to influenza in children between 0 and 4 years of age and between 5 and 14 years of age than in the six pre-pandemic seasons (not shown).

## 4. Discussion

The present analysis compiles data not only for SARS-CoV-2 but also for the other major respiratory pathogens to investigate how the epidemiology of respiratory illnesses in children has changed in the three pandemic years from 2020– 2022. Children’s ARI rates remained almost consistently below the ARI rates that would have been expected based on previous years over a period of approximately 1.5 years after the start of the pandemic. While rhinoviruses continued to lead to ARI after a short break, the usual wave of influenza occurring in the first three to four months of the year was absent in winter 2020/2021, as was the RSV wave. In autumn 2022, there was an almost simultaneous, premature RSV and influenza wave leading to very high ARI rates. COVID-19 did not lead to measurable morbidity in children at population level until the emergence of the Omicron variant (from 2022), but even then the hospitalisation rate remained comparatively low.

Since the beginning of the pandemic the course of the ARI rate in children differed substantially in almost all phases from the course known from pre-pandemic years. The greatest similarities in terms of trend and incidence were observed for just a few weeks during the usual low-incidence phases in summer 2020 and 2021. The at times extensive contact reduction measures (during the first lockdown including school closures, or, during the second lockdown including school closures, split and alternating class attendance, systematic testing in schools (and day-care centres), wearing of masks and the spatial or temporal separation of groups) and the responsible behaviour in the population effectively reduced ARI rates and thus the transmission of any respiratory pathogens over an extended period of time.

Initially (from around May/June 2020), rhinoviruses were the only pathogens that still seemed to cause respiratory illness. This was analysed and described early on in a more detailed review [8]. Possible explanations that were discussed included that rhinoviruses are transmitted more than other viruses via fomite and contact infection or also that masks prevent the transmission of rhinoviruses via droplets or aerosol less effectively than other viruses [8]. The almost complete ‘suppression’ of RSV and influenza virus transmission in the winter of 2020/2021 resulted in an increasing proportion of susceptible, immune naive young children over time. In the two RSV waves in 2021 and 2022, the proportion of children between 0 and 4 years of age admitted as inpatients with an RSV diagnosis (2021: 1.1%; 2022: 0.9%) was about twice the average of the pre-pandemic waves in the years between 2014/2015 and 2019/2020 (average 0.5%). The extent to which the increased incidence of RSV illnesses had an impact on the number of very severe courses, especially cases of bronchiolitis in infants, needs to be explored by further analyses. In France, the RSV wave shifted as well, but started already in spring 2021 and the peak of the disease was not as high as usual [28, 29]. An increased unusually timed circulation of influenza virus in May 2022 failed to build up to an influenza wave to a degree as commonly observed. Likewise, the next RSV wave starting in October 2022 in Germany began unusually early. This was also consistent with the results from several European countries [30]. An aggravating factor – consistent with the scenario in Germany – was the parallel and very early co-circulation of (RSV and) influenza viruses [30]. In Germany, the influenza wave at the end of 2022 led to a higher influenza-related SARI rate in both age groups of children than in the entire six pre-pandemic seasons. In summary, it can be concluded that, especially after the absence of RSV waves and influenza waves, stronger and earlier waves occurred compared to pre-pandemic years. As these waves also occurred – at least at the end of 2022 – simultaneously, this led to hardly manageable challenges for the health care system [31].

It is evident from our analyses that the incidence of respiratory illnesses depends above all on age and season. On average, young children experience approximately 3–4 ARI in the winter half-year, and approximately 2.5 in the summer half-year, which is still more than are experienced by adults in the winter half-year [1, 3]. Even after the pronounced decline in ARI at the beginning of the pandemic (sixth column group in Figure 4), the average number of ARI rose again in the following six months, first in infants, then in older children and at last in adults. In contrast, at the beginning of each COVID-19 wave, the highest incidence of COVID-19 cases reported to the RKI (via the mandatory reporting system) was consistently observed in adolescents or young adults first and only in the weeks thereafter in the neighbouring age groups, i.e. older adults and (young) children [32–35]. Thus, after the third year after its appearance COVID-19 has not (yet) developed into a disease that – unlike many other infectious respiratory diseases – receives its impulse and momentum from child age groups.

The incidence of viral co-infections was particularly high in infants in the analysed period and decreased clearly after the age of 5 years (Figure 5). Similar findings were also observed prior to the COVID-19 pandemic, e.g. by Mandelia et al. [36] detecting an even more pronounced difference between under-18-year-olds and adults (factor of 7 [36] vs. factor of 3 in our results) in an analysis of data from 2013–2018. However, this might be related to the different time periods (with different pathogen incidences) and boundary conditions. It is interesting to note that the actual co-infection incidence in this age group does not always correspond to the expected incidence, i.e. extensive co-circulation of two pathogens does not mean that dual infections by these two pathogens occur as frequently as expected. There could be many reasons for the fact that the combination of rhinovirus + RSV in 0- to 4-year-olds during the last quarter of 2021 roughly corresponds to the expected incidence (Figure 6), but the combination of influenza + RSV, for example, falls clearly short (Figure 7). It is possible that rhinovirus or RSV infections are generally not severe enough that the affected child is excluded from the social network for an extended period of time and is thus almost continuously exposed to the infection pressure of the second pathogen as well. Conversely, it is possible that the regional distribution is inhomogeneous, and finally, blocking mechanisms at the cellular level might also play a role [37, 38]. Greer [26] had indicated that RSV and rhinovirus infections are significantly less frequent than expected, however, he also found that the negative correlation was much weaker when the analysis was restricted to certain age groups, e.g. infants. Therefore, we limited our analyses to 0- to 4-year-old children from the very beginning. A relevant question is whether or not children with co-infections also have more severe courses of disease. In that regard a systematic review in 2020 of publications up to 2019 investigating co-infections with RSV found no evidence for more severe courses of disease, although co-infections by RSV and hMPV may be an exception [39].

Since the onset of the COVID-19 pandemic, there has been evidence that the severity of the COVID-19 disease was clearly lower in children than in (older) adults [40]. Scientific studies suggest that this is related to innate immunity, which responds immediately to the virus at the local (anatomical) level, for example through cells of the nasal mucosa [41, 42]. Cross-protection by infection with other human coronaviruses that have been circulating seasonally for a long time, e.g. NL63, HKU1 or OC-43, which frequently affect children under five years of age, has also been suggested [43, 44]. Although the susceptibility of children to SARS-CoV-2 infection (measured, for example, via the household attack rates) increased from the Alpha variant onwards [45, 46], it was not until the phases of the Delta (b) wave (in the second half of 2021) and during the Omicron BA.1 and BA.2 waves (in the first four months of 2022) that there was a significant increase at population-level reaching a low percentage level of SARS-CoV-2 infections in children with ARI symptoms. Nevertheless, the comprehensive testing of children in daycare and school settings conducted in the second half of 2021 and first half of 2022 may have resulted in children with COVID-19 visiting a doctor’s office less frequently. This, in turn, may have caused the proportion of children with COVID-19 in the virological surveillance sample to be underrepresented and consequently it is possible that we underestimated the COVID-ARI rate. In addition, especially in children, the symptoms were sometimes so mild that the ARI definition used in GrippeWeb (experience of cough, sore throat or fever) was not met. As a result, the COVID-ARI rate did not include asymptomatic children, but also children with very mild COVID-19 symptoms were not taken into consideration. Asymptomatic SARS-CoV-2 infections were particularly common in children: according to one review article, the proportion of asymptomatic infections was highest in school-age children (at 36%) and steadily decreased with older ages [47]. Indeed, estimates from a Bavarian seroprevalence study [48] in children and adolescents aged 1–17 years at the end of 2021 and after the first half of 2022 were 20% and 74%, respectively, which is clearly higher than our calculated cumulative COVID-ARI rates in children of 6% and 38%, respectively, at the same points in time. In adults, on the other hand, a seroprevalence of anti-spike antibodies of at least 90% was already detectable towards the end of 2021 [49]. Antibodies against the spike protein are produced by the immune system either after an infection or after a vaccination. It was interesting to note that as early as during the two Omicron BA.5 waves (that followed the BA.1 and BA.2 waves) the COVID-ARI rate was again higher in adults than in children. This suggests that (possibly multiple) exposures of the immune system to the virus or its components (antigens) had led to a stronger or longer lasting immune reaction in children than in adults.

## Limitations

A limitation of our analyses is that the participants in GrippeWeb can generally be considered to be rather healthsavvy. Nevertheless, it has been shown that comparable data from GrippeWeb were – also quantitatively – quite consistent with those from the sentinel surveillance system using data from primary care physicians (Working Group for Influenza (Arbeitsgemeinschaft Influenza)) and can therefore be considered trustworthy [50]. Another possible limitation is the calculation of pathogen-specific ARI rates is based on the pathogens detected in virological surveillance. Thus, data from two systems (firstly, ARI consultations in the outpatient setting, and secondly, the GrippeWeb population reporting data independent of a doctor’s visit) are combined. However, we took into account the differences that may arise from different consultation behaviour of individuals affected by ILI and non-ILI by adjusting for ILI and non-ILI rates. This allowed us to calculate population-based estimates of pathogen-specific ARI rates. A third limitation is that the calculation of the expected incidences of co-infections assumes that infections with the individual pathogens occur independently of each other and are evenly distributed throughout Germany. As a fourth limitation, it could be mentioned that the cumulative SARI rates due to certain pathogens do not include information on vaccinations. However, this was not intended as the rates shown here are meant to reflect exactly what occurs in the population regardless of vaccination.

## Conclusion

Respiratory infections occurred much less frequently than usual in children during the COVID-19 pandemic as a result of contact-reducing and transmission-inhibiting measures over a period of approximately 1.5 years. Whereas rhinoviruses spread continuously again after a short period of time, the RSV and influenza waves did not occur in the autumn-winter season of 2020/2021. The (almost simultaneous) RSV and influenza wave in the last quarter of 2022 started earlier and were stronger than was common before the pandemic. Whether this ‘catch-up effect’ reduced the incidence of particularly severe manifestations, especially of bronchiolitis in infants, must be investigated by further, more in-depth analyses and, if necessary, through the inclusion of other systems. Only after the emergence of the Omicron variant did SARS-CoV-2 lead to infection in about half of the children and this occurred over a short period of time, but even then illnesses requiring hospitalisation remained rare compared to RSV or influenza waves. Future developments will show whether COVID-19 will become a ‘children’s disease’ or if the highest incidences and more severe illnesses will occur primarily in adults.

## Key statements

- The measures taken during the COVID-19 pandemic and the responsible behaviour of the population led to low rates of ARI in children.
- In the winter of 2020/2021, the usual influenza wave normally occurring in the first three to four months of the year was absent, as was the RSV wave.
- In autumn 2022, the RSV and influenza waves led to exceptionally high ARI rates in children.
- In the period studied (2020–2022) a quarter of the children experienced a respiratory illness due to rhinovirus followed by influenza viruses and RSV.
- COVID-19 did not lead to measurable morbidity in children at population level until the emergence of the Omicron variant (from 2022), but even then the hospitalisation rate remained comparatively low.

## Figures and Tables

**Table 1:** Proportion of all ARI due to different respiratory pathogens in 0- to 14-year-olds; sorted in descending order (period 2020−2022; proportion adjusted for ILI and non-ILI) Source: GrippeWeb and Virological Surveillance of the Working Group for Influenza (Arbeitsgemeinschaft Influenza)

Pathogen	0–14 years of age
Rhinovirus	25,3%
Influenza (all)	11,5%
RSV	10,5%
Influenza A(H3N2)	8,9%
Parainfluenza virus	8,1%
hMPV	4,4%
SARS-CoV-2	4,4%
Coronavirus OC43	2,7%
Coronavirus NL63	2,2%
Influenza A(H1N1)	1,5%
Influenza B	1,1%
Coronavirus HKU1	0,9%
Coronavirus 229E	0,4%
